# Severe persistent hypocholesterolemia after emergency gastrointestinal surgery predicts in-hospital mortality in critically ill patients with diffuse peritonitis

**DOI:** 10.1371/journal.pone.0200187

**Published:** 2018-07-06

**Authors:** Seung Hwan Lee, Jin Young Lee, Tae Hwa Hong, Bo Ok Kim, Yeon Ju Lee, Jae Gil Lee

**Affiliations:** 1 Department of Surgery, Yonsei University College of Medicine, Seoul, Republic of Korea; 2 Trauma Training Center, Severance Hospital, Yonsei University Health System, Seoul, Republic of Korea; 3 Department of Trauma Surgery, Trauma Center, Chungbuk National University Hospital, Cheongju, Republic of Korea; 4 Department of Surgery, Hallym University Sacred Heart Hospital, Hallym University College of Medicine, Anyang, Republic of Korea; 5 Department of Research Affairs, Biostatistics Collaboration Unit, Yonsei University College of Medicine, Seoul, Republic of Korea; Indiana University, UNITED STATES

## Abstract

**Background:**

Plasma cholesterol acts as a negative acute phase reactant. Total cholesterol decreases after surgery and in various pathological conditions, including trauma, sepsis, burns, and liver dysfunction. This study aimed to determine whether hypocholesterolemia after emergency gastrointestinal (GI) surgery is associated with in-hospital mortality in patients with diffuse peritonitis.

**Methods:**

The medical records of 926 critically ill patients who had undergone emergency GI surgery for diffuse peritonitis, between January 2007 and December 2015, were retrospectively analyzed. The integrated areas under the curve (iAUCs) were calculated to compare the predictive accuracy of total cholesterol values from postoperative days (PODs) 0, 1, 3, and 7. Cox proportional hazard regression modeling was performed for all possible predictors identified in the univariate and multivariable analyses.

**Results:**

The total cholesterol level measured on POD 7 had the highest iAUC (0.7292; 95% confidence interval, 0.6696–0.7891) and was significantly better at predicting in-hospital mortality than measurements on other days. The optimal total cholesterol cut-off value for predicting in-hospital mortality was 61 mg/dL and was determined on POD 7. A Cox proportional hazard regression analysis revealed that a POD 7 total cholesterol level < 61 mg/dL was an independent predictor of in-hospital mortality after emergency GI surgery (hazard ratio, 3.961; 95% confidence interval, 1.786–8.784).

**Conclusion:**

Severe persistent hypocholesterolemia (<61 mg/dL) on POD 7 independently predicted in-hospital mortality, after emergency GI surgery, in critically ill patients with diffuse peritonitis.

## Introduction

In complicated intra-abdominal infections (cIAIs), the infectious process extends beyond the organ and may cause either localized or diffuse peritonitis [1]. In particular, diffuse peritonitis is an important cause of morbidity and may be associated with a poor prognosis. The early prognostic evaluation and detection of diffuse peritonitis are crucial for the assessment of disease severity and the delivery of optimal treatment [2]. Many factors that influence the prognoses of patients with diffuse peritonitis have been described, including advanced age, poor nutritional status, pre-existing illness, immunosuppression, extended peritonitis, presence of septic shock, inadequate source control, organ failure, and nosocomial infection [2–9]. Moreover, a recent report by Suarez-de-la-Rica et al. described an investigation of certain biomarkers (procalcitonin, C reactive protein, and lactate) as predictors of mortality in surgical patients with diffuse peritonitis [10].

However, the use of biochemical parameters as predictors of clinical outcomes in diffuse peritonitis has not yet been sufficiently studied. Some biochemical parameters, directly or indirectly, reflect the host response and infection severity. In 1926, Thannhauser and Schaber reported an association between low cholesterol levels and disease [11]. Subsequently, total cholesterol levels have been shown to be substantially reduced in various pathological conditions, including infections, burns, and cancer. Although hypocholesterolemia is anecdotally considered as a marker of malnutrition, plasma cholesterol levels are known to decrease after surgery, trauma, and acute hemorrhage, as well as during sepsis and liver dysfunction [12–15]. Several studies have specifically focused on the relationship between low cholesterol levels and sepsis [16–18]. However, the critical cholesterol level and the association of hypocholesterolemia duration with mortality in surgical patients with diffuse peritonitis remain unclear.

The present study investigated the association between hypocholesterolemia after emergency gastrointestinal (GI) surgery and in-hospital mortality among patients with diffuse peritonitis.

## Materials and methods

### Patients

**Fig 1:**
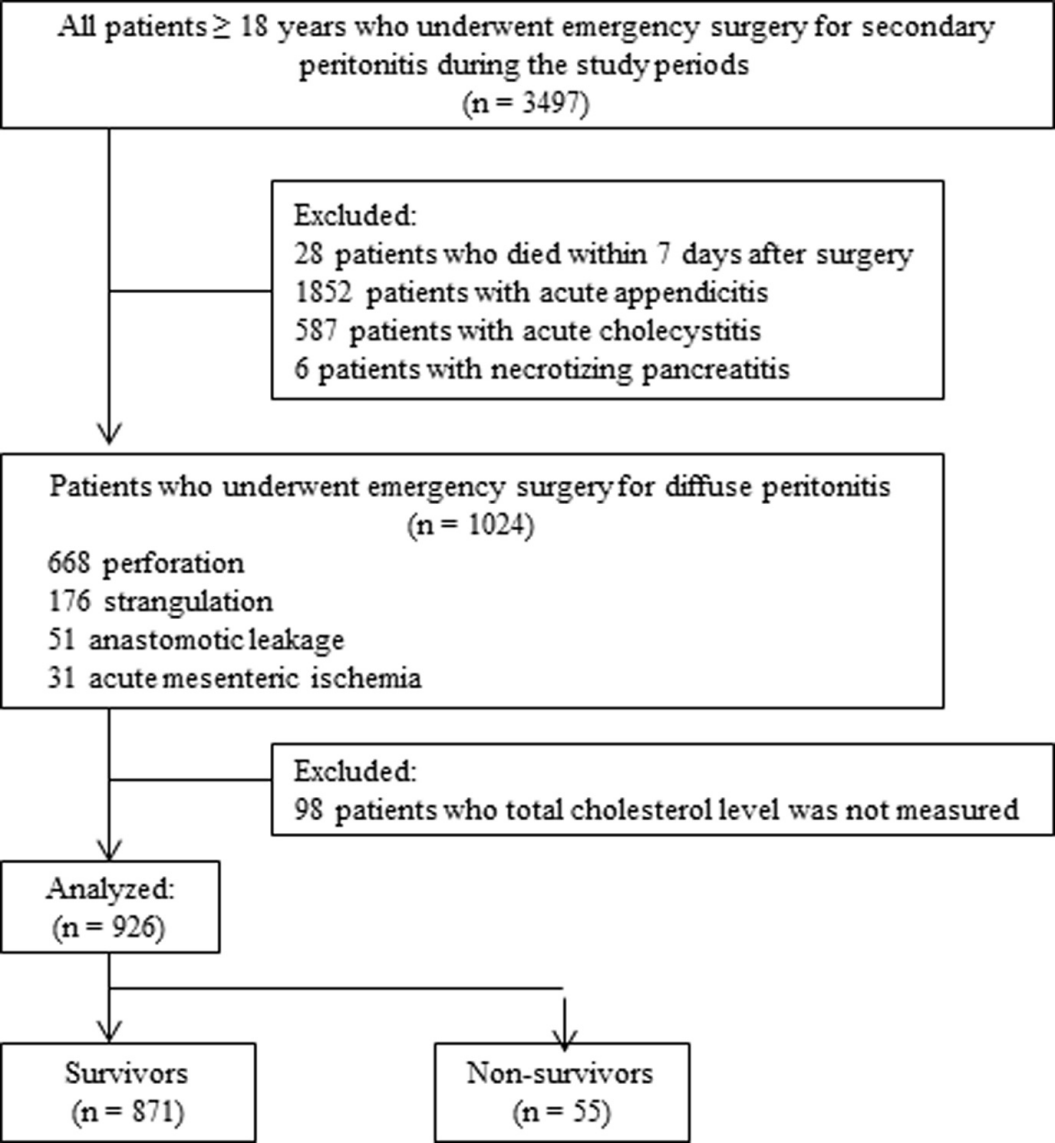
Flow diagram of the patient selection process.

The medical records of critically ill patients (≥18 years old) who had undergone emergency surgery for diffuse peritonitis at Severance Hospital, Yonsei University College of Medicine, Seoul, Republic of Korea, between January 2007 and December 2015, were retrospectively reviewed. Patients with diffuse peritonitis due to GI perforation, anastomotic leakage, intestinal strangulation, and acute mesenteric ischemia were included. Patients who had undergone surgery for acute appendicitis, acute cholecystitis, or necrotizing pancreatitis were excluded. Most patients with acute appendicitis or acute cholecystitis were excluded due to the presence of localized peritonitis, and patients with necrotizing pancreatitis requiring repeated surgery were excluded to maintain study population homogeneity. We also excluded patients who died within the first 7 days after surgery. Finally, we excluded patients with total cholesterol levels that were not measured before surgery, immediately after surgery, or on postoperative days (PODs) 1, 3, or 7 (Fig 1). This study was approved by the Severance Institutional Review Board (IRB No. 4-2016-0177), which waived the requirement for informed consent due to the retrospective nature of the study.

### Perioperative variables

The following variables were included in the analysis: age, sex, body mass index, American Society of Anesthesiologists physical status classification, Acute Physiology and Chronic Health Evaluation II (APACHE II) score, comorbidities, lesion location, diagnosis, perioperative shock, preoperative blood cultures, intraoperative peritoneal fluid cultures, preoperative laboratory findings, postoperative complications, and surgery type. Perioperative shock was septic shock occurring before, during, or after surgery, and was defined as sepsis-induced hypotension persisting despite adequate fluid resuscitation [19]. All surgeries were performed within a maximum of 12 hours after diagnosis. Pulmonary complications were defined as the presence of one or more of the following postoperative conditions: pneumonia, atelectasis, pleural effusion, or acute respiratory distress syndrome. Anastomotic leakage was defined as clinical signs of leakage, such as the presence of a fecaloid drain; emission of fecal material from the wound; contrast extravasation during an enema; evidence of postoperative peritonitis during surgery; or fluid collection or air in the anastomotic region, as seen using computed tomography. Wound complications were defined as the presence of a wound infection, including superficial incisional surgical site infection (SSI); deep incisional SSI; organ/space SSI, according to the definition proposed by the Centers for Disease Control and Prevention (Atlanta, GA, USA); or wound dehiscence [20]. Postoperative acute kidney injury (AKI) was defined according to the Risk, Injury, Failure, Loss and End-Stage Renal Disease (RIFLE) definition, using only serum creatinine changes (i.e., without urine output criteria). RIFLE defines AKI based on a ≥ 50% change in serum creatinine levels from a reference value, while also accounting for patients with documented chronic kidney disease at the time of admission. Postoperative ileus was defined as a non-mechanical obstruction lasting > 5 days or reinsertion of the nasogastric tube within the first 5 days, and was confirmed using simple abdominal radiography. Newly developed sepsis was defined as sepsis diagnosed in patients who did not initially demonstrate sepsis or septic shock or in patients who were again diagnosed with sepsis during a period of stability or improvement after a previous septic episode (e.g., sepsis, severe sepsis, or septic shock).

### Laboratory variables

Among the collected laboratory variables, total cholesterol levels (reference range, 142–240 mg/dL) measured before surgery, immediately after surgery, and on PODs 1, 3, and 7 were analyzed.

### Outcome parameters

The primary outcome investigated using Cox regression analysis was in-hospital mortality, which was defined as death occurring during hospitalization after the emergency GI surgery.

### Statistical analysis

Prior to the statistical analysis, data normality was tested using the Shapiro–Wilk test. Categorical variables are presented as numbers (%) and were compared, between groups, using the chi-square test. Continuous variables are expressed as means ± standard deviations and were compared, between groups, using Student’s *t*-test. Any differences in the total cholesterol levels between survivors and nonsurvivors were evaluated using repeated measures analysis of variance (ANOVA), followed by Tukey`s post-hoc test. The time-dependent receiver operating characteristic (ROC) curve method was used to compare the predictive accuracy of the total cholesterol measurements, each day, on in-hospital mortality [21]. We compared the integrated areas under the curve (iAUCs) for each total cholesterol measurement. The iAUC is a weighted average of areas under the curve across the follow-up period and is a measure of the predictive accuracy of the total cholesterol level on each measurement day during follow-up; higher iAUCs indicate better predictive accuracy. Differences in iAUC values across the five total cholesterol measurement days were calculated using a bootstrapping method, resampling 1000 times. The Contal and O’Quigley method, based on the log-rank test, was used to determine the cut-off value for the most useful cholesterol level predicting in-hospital mortality [22]. Survival curves were constructed using the Kaplan–Meier method and compared using the log-rank test. A Cox proportional hazards model was used to investigate predictors of in-hospital mortality. Factors with a *P-*value < 0.05 in the univariate Cox regression analysis were considered as potential predictors, and were selected for the multivariable regression analysis. The hazard ratios (HRs) are shown with 95% confidence intervals (CIs), and *P*-values < 0.05 were considered statistically significant. Statistical analyses were conducted using SPSS version 20.0.0 (IBM; Armonk, NY, USA), SAS version 9.2 (SAS Institute; Cary, NC, USA), and R version 3.1.3 (http://www.R-project.org/).

## Results

A total of 3497 adult patients underwent emergency surgery for secondary peritonitis, between January 2007 and December 2015, and were screened for this analysis; 926 were included in the present study. The mean patient age was 59.7 ± 16.1 years; 581 patients (62.7%) were male. A total of 83 patients (8.7%), including 28 patients who died within the first 7 days, died during the postoperative hospital stay. The mean time between surgery and death was 26.0 ± 18.7 days. The most common cause of diffuse peritonitis was perforations (72.1%) of the GI tract, and predominantly resulted from pathological changes in the large (colon and rectum, 44.0%) and small (40.8%) bowels. Preoperative total cholesterol levels were significantly higher in survivors than in nonsurvivors (138.3 ± 48.3 mg/dL versus 106.0 ± 48.8 mg/dL, *P* < 0.001). The primary surgical procedures were primary repair (25.5%), small bowel resection with anastomosis (23%), enterostomy (15.8%), colostomy (15.8%), colorectal resection with anastomosis (14.6%), and subtotal or total gastrectomy (5.4%). Other baseline characteristics are shown in Table 1.

**Table 1:** Baseline characteristics of the total population.^a^

Characteristic	Total (n = 926)	Survivors (n = 871)	Non-survivors (n = 55)	*P*-value
Age, mean (SD), y	59.7 ± 16.1	59.6 ± 16.2	62.1 ± 14.6	0.246
Sex				
Male	581 (62.7)	548 (62.9)	33 (60.0)	0.664
Female	345 (37.3)	323 (37.1)	22 (40.0)	
BMI, mean (SD), kg/m^2^	21.9 ± 3.5	22.0 ± 3.5	21.5 ± 3.5	0.286
Comorbidity				
Hypertension	307 (33.2)	285 (32.7)	22 (40.0)	0.266
DM	131 (14.1)	123 (14.1)	8 (14.5)	0.93
CRF	51 (5.5)	41 (4.7)	10 (18.2)	<0.001
Pulmonary tuberculosis	61 (6.6)	58 (6.7)	3 (5.5)	0.727
Malignancy	485 (52.4)	449 (51.5)	36 (65.5)	0.045
ASA score	2.0 ± 0.9	2.0 ± 0.9	2.4 ± 1.2	0.03
APACHE II score	20.7 ± 8.2	19.9 ± 7.8	27.1 ± 8.8	<0.001
Location of lesion				
Stomach	141 (15.2)	133 (15.3)	8 (14.5)	0.957
Duodenum	61 (6.6)	58 (6.7)	3 (5.5)	
Jejunum and ileum	317 (34.2)	299 (34.3)	18 (32.7)	
Colon and rectum	407 (44.0)	381 (43.7)	26 (47.3)	
Diagnosis				
Perforation	668 (72.1)	626 (71.9)	42 (76.4)	0.316
Strangulation	176 (19.0)	170 (19.5)	6 (10.9)	
Anastomotic leakage	51 (5.5)	46 (5.3)	5 (9.1)	
Acute mesenteric ischemia	31 (3.3)	29 (3.3)	2 (3.6)	
Perioperative shock	226 (24.4)	186 (21.4)	40 (72.7)	<0.001
Type of procedure				
Primary repair	236 (25.5)	224 (25.7)	12 (21.8)	0.783
Small bowel resection with anastomosis	213 (23.0)	201 (23.1)	12 (21.8)	
Ileo- or jejunostomy	146 (15.8)	134 (15.4)	12 (21.8)	
Hartmann`s procedures or colostomy	146 (15.8)	136 (15.6)	10 (18.2)	
Colon resection with anastomosis	135 (14.6)	129 (14.8)	6 (10.9)	
Gastrectomy (subtotal or total)	50 (5.4)	47 (5.4)	3 (5.5)	
Culture positive				
Preoperative blood	46 (8.8)	38 (7.9)	8 (20.5)	0.008
Intraoperative peritoneal fluid	271 (63.9)	232 (61.5)	39 (83.0)	0.004
Preoperative laboratory findings				
Hemoglobin, mean (SD), g/dL	11.9 ± 2.5	12.1 ± 2.4	10.5 ± 2.3	<0.001
Platelets, mean (SD), 10^3^/μL	269.3 ± 132.0	274.2 ± 129.9	196.5 ± 142.7	<0.001
Albumin, mean (SD), g/dL	3.4 ± 0.7	3.4 ± 0.7	2.7 ± 0.6	<0.001
Total bilirubin, mean (SD), mg/dL	1.2 ± 1.0	1.0 ± 0.9	1.5 ± 1.2	0.006
Total cholesterol, mean (SD), mg/dL	136.1 ± 49.0	138.3 ± 48.3	106.0 ± 48.8	<0.001
CRP, mean (SD), mg/L	96.3 ± 90.6	95.8 ± 89.1	111.3 ± 102.8	0.257
Postoperative complications				
Anastomotic leakage	55 (5.9)	42 (4.8)	13 (23.6)	<0.001
Wound complication	185 (20.0)	170 (19.5)	15 (27.3)	0.163
Newly developed sepsis	59 (6.4)	30 (3.4)	29 (52.7)	<0.001
Pulmonary complication	187 (20.2)	150 (17.2)	37 (67.3)	<0.001
Acute kidney injury	124 (13.4)	93 (10.7)	31 (56.4)	<0.001
Ileus	132 (14.3)	116 (13.3)	16 (29.1)	0.001

SD, standard deviation; DM, diabetes mellitus; CRF, chronic renal failure; ASA, American Society of Anesthesiologists physical status classification; APACHE II, Acute Physiology and Chronic Health Evaluation II; CRP, C-reactive protein.

^a^ Data are presented as numbers (percentage) of patients unless otherwise indicated.

**Fig 2:**
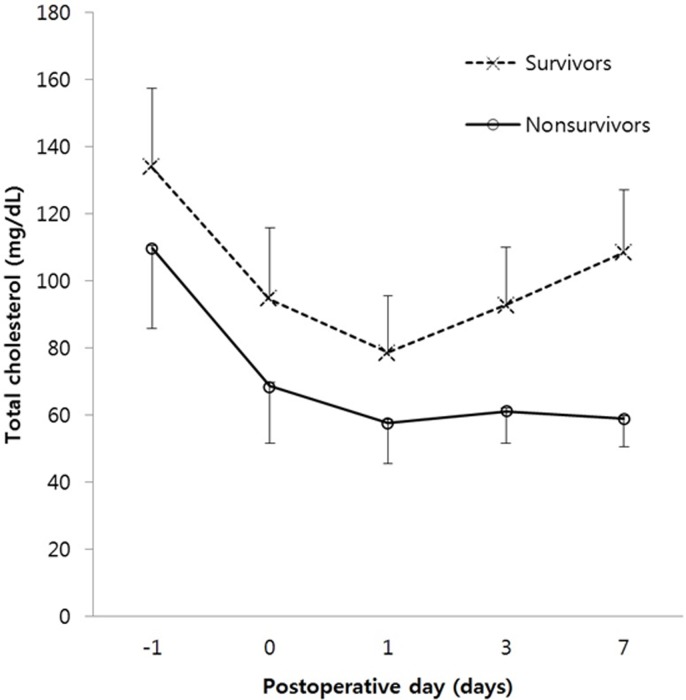
Comparison of the changes in total cholesterol levels between survivors and nonsurvivors. The time variations in total cholesterol levels were significantly different between survivors and nonsurvivors (Greenhouse-Geisser corrected, *P* < 0.001, using repeated-measures analysis of variance).

**Fig 3:**
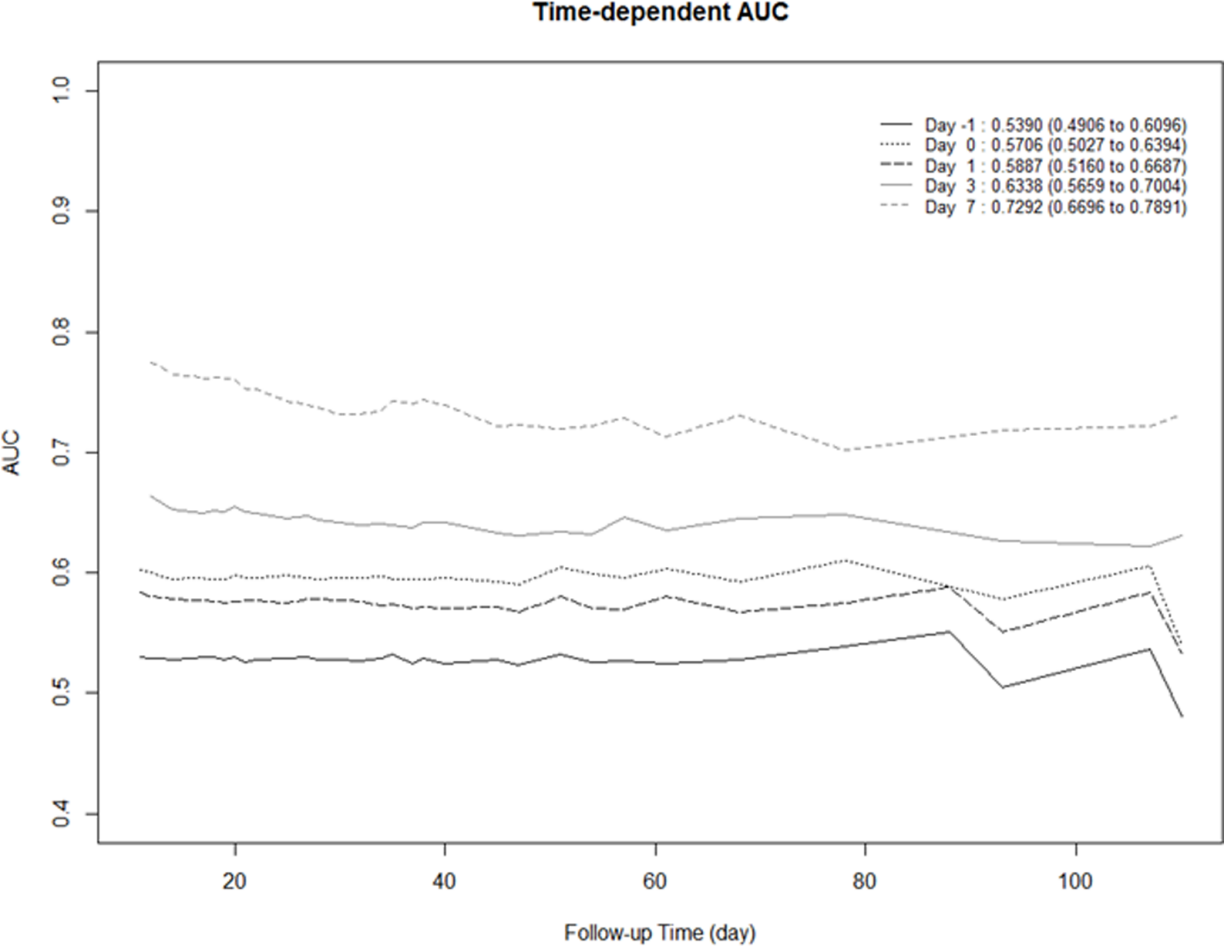
Time-dependent receiver operating curve analysis to evaluate the predictive accuracy of each measurement day. Results in the top right-hand corner of the figure indicate the integrated area under the curve (iAUC) and 95% confidence interval. The iAUC is a measure of the predictive accuracy for in-hospital mortality on each measurement day. The cholesterol level measured on postoperative day 7 had the highest iAUC.

Among nonsurvivors, total cholesterol levels decreased markedly on POD 1 and continued to gradually decrease until POD 7. However, among survivors, total cholesterol levels only decreased until POD 1 and then gradually increased until POD 7. Repeated-measures ANOVA indicated that the changes in total cholesterol level over time were significantly different between the two groups (Greenhouse-Geisser corrected, *P* < 0.001; Fig 2). The results of the time-dependent ROC curve analysis, over the course of the hospital stay, are shown in Fig 3. Of the 5 measurement days, the total cholesterol levels measured on POD 7 had the highest iAUC value; the iAUC values were significantly different between POD 7 and all other days (day before surgery, immediately after surgery, POD 1, and POD 3).

The estimated differences in iAUCs, relative to POD 7, were -0.1902 (95% CI, -0.2691 to -0.0966, preoperative), -0.1586 (95% CI, -0.2525 to -0.0658, immediately after surgery), -0.1404 (95% CI, -0.2350 to -0.0421, POD 1), and -0.0954 (95% CI, -0.1870 to -0.0055, POD 3), indicating significantly better prediction of in-hospital mortality using cholesterol values measured on POD 7 than for any other day (Table 2). Thereafter, using the Contal and O’Quigley method, the optimal POD 7 total cholesterol cut-off value for predicting in-hospital mortality was 61 mg/dL.

**Table 2:** Estimated iAUC differences using a bootstrapping method.

Difference	iAUC (95% CI)
Pre-OP versus POD 0	-0.0316 (-0.1175 to 0.0600)
Pre-OP versus POD 1	-0.0497 (-0.1454 to 0.0490)
Pre-OP versus POD 3	-0.0948 (-0.1830 to -0.0023)
Pre-OP versus POD 7	-0.1902 (-0.2691 to -0.0966)
POD 0 versus POD 1	-0.0182 (-0.1194 to 0.0856)
POD 0 versus POD 3	-0.0632 (-0.1639 to 0.0349)
POD 0 versus POD 7	-0.1586 (-0.2525 to -0.0658)
POD 1 versus POD 3	-0.0451 (-0.1399 to 0.0616)
POD 1 versus POD 7	-0.1404 (-0.2350 to -0.0421)
POD 3 versus POD 7	-0.0954 (-0.1870 to -0.0055)

Pre-OP, preoperative; POD, postoperative day; CI, confidence interval; iAUC, integrated area under the curve.

**Fig 4:**
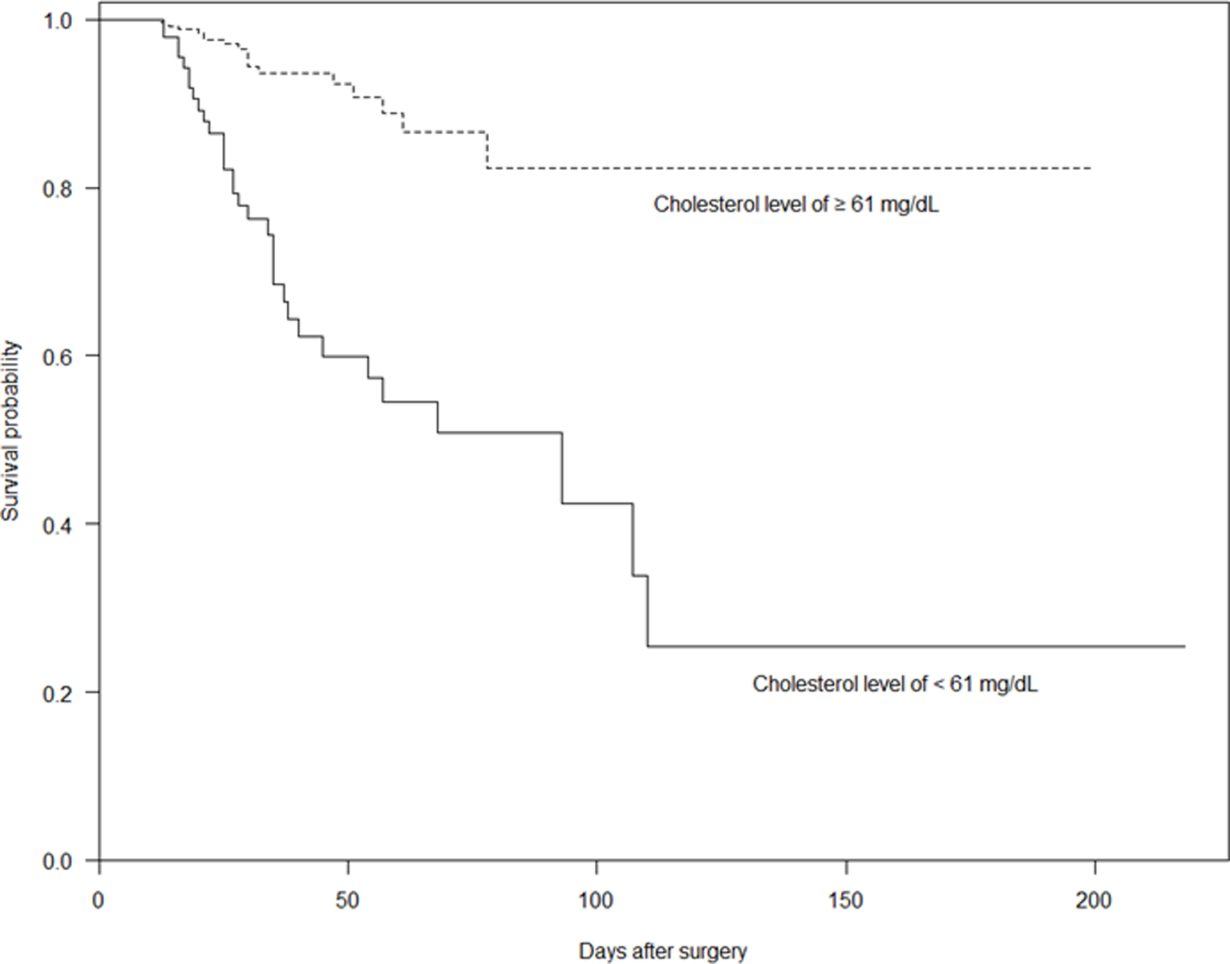
Kaplan–Meier curve based on hypocholesterolemia severity (total cholesterol level ≥61 mg/dL versus total cholesterol level <61 mg/dL; log-rank test, *P* < 0.0001).

The cumulative patient survival, stratified by total cholesterol level ≥61 mg/dL and <61mg/dL on POD 7, is shown in Fig 4; the cumulative survival of patients with a total cholesterol level of ≥61 mg/dL on POD 7 was significantly better than that of those with a total cholesterol level of <61 mg/dL (*P* < 0.0001).

Perioperative variables, including the POD 7 total cholesterol level, were assessed for association with in-hospital mortality using univariate and multivariable Cox regression analyses. The results revealed that a total cholesterol level < 61 mg/dL on POD 7 (HR, 3.961; 95% CI, 1.786–8.784), a lower platelet count before surgery, and the presence of newly developed sepsis were independent predictors of in-hospital mortality after emergency GI surgery (Table 3).

**Table 3:** Univariate and multivariable Cox proportional hazards model for in-hospital mortality.

Characteristics	Univariate analysis		Multivariate analysis	
	HR (95% CI)	*P*-value	HR (95% CI)	*P*-value
Age	1.009 (0.991–1.027)	0.3310		
Sex				
Female	Ref.			
Male	1.000 (0.582–1.719)	>0.9999		
BMI	0.977 (0.911–1.047)	0.5104		
Comorbidity	2.003 (1.001–4.008)	0.0495		
Hypertension	1.040 (0.605–1.787)	0.8872		
DM	0.708 (0.334–1.503)	0.3688		
CRF	2.003 (1.001–4.008)	0.0495	1.462 (0.492–4.345)	0.4946
Pulmonary tuberculosis	0.627 (0.196–2.009)	0.4319		
Malignancy	1.337 (0.766–2.333)	0.3065		
ASA score	1.151 (0.902–1.469)	0.2586		
APACHE II score	1.071 (1.036–1.108)	<0.0001	1.024 (0.977–1.074)	0.3139
Location of lesion				
Stomach	Ref.			
Duodenum	0.662 (0.175–2.500)	0.5429		
Jejunum and ileum	0.701 (0.305–1.613)	0.4034		
Colon and rectum	0.786 (0.356–1.737)	0.5515		
Diagnosis				
Perforation	Ref.			
Strangulation	0.709 (0.301–1.670)	0.4318		
Anastomotic leakage	0.588 (0.142–2.436)	0.4641		
Acute mesenteric ischemia	1.056 (0.415–2.685)	0.9091		
Perioperative shock (t) ^a^	0.348 (0.126–0.958)	0.0411	1.250 (0.975–1.603)	0.0783
Culture positive				
Preoperative blood	2.624 (1.199–5.742)	0.0158	1.878 (0.708–4.984)	0.2054
Intraoperative peritoneal fluid	1.366 (0.636–2.934)	0.4241		
Preoperative laboratory findings				
Hemoglobin	0.935 (0.825–1.059)	0.2896		
Platelet	0.996 (0.994–0.999)	0.0016	0.997 (0.994–1.000)	0.0389
Albumin	0.520 (0.316–0.854)	0.0098	1.200 (0.669–2.155)	0.5405
Total bilirubin	1.074 (0.933–1.237)	0.3222		
CRP	1.001 (0.997–1.004)	0.7466		
POD 7 total cholesterol level <61 mg/dL	5.228 (2.949–9.268)	<0.0001	3.961 (1.786–8.784)	0.0007
Postoperative complications				
Anastomotic leakage	1.463 (0.772–2.772)	0.2433		
Wound complication	0.787 (0.434–1.427)	0.4297		
Newly developed sepsis (t) ^a^	0.345 (0.136–0.876)	0.0251	1.344 (1.074–1.682)	0.0098
Pulmonary complication (t) ^a^	0.308 (0.117–0.812)	0.0173	1.272 (0.985–1.643)	0.0650
Acute kidney injury	3.540 (2.065–6.069)	<0.0001	1.509 (0.587–3.874)	0.3928
Ileus	1.714 (0.955–3.076)	0.0710		
Type of procedure				
Primary repair	Ref.			
Small bowel resection with anastomosis	1.174 (0.329–4.190)	0.8045		
Ileo- or jejunostomy	1.222 (0.545–2.738)	0.6264		
Hartmann`s procedures or colostomy	1.148 (0.430–3.068)	0.7825		
Colon resection with anastomosis	0.798 (0.345–1.850)	0.5993		
Gastrectomy (subtotal or total)	0.978 (0.438–2.183)	0.9564		

DM, diabetes mellitus; CRF, chronic renal failure; ASA, American Society of Anesthesiologists physical status classification; APACHE II, Acute Physiology and Chronic Health Evaluation II; POD, postoperative day; CRP, C-reactive protein.

^a^ These are time-dependent variables that were used in the univariate and multivariable Cox regression model.

## Discussion

cIAIs are important causes of morbidity and poor clinical prognoses. In a recent study, the overall mortality rate for patients with cIAIs was 10.1% (4% in patients with localized peritonitis and 18% in patients with diffuse peritonitis) [1]. In the present study, the mortality rate for patients with diffuse peritonitis was 8.7%. Despite advances in diagnostic tools, surgical management, intensive medical care, and antimicrobial therapy, the prognosis for patients with diffuse peritonitis remains poor.

For this reason, over the past three decades, several scoring systems have been developed to determine the prognosis of patients with peritonitis, including the Mannheim Peritonitis Index (MPI) [23]. Prognostic factors have also been investigated as predictors of mortality in patients with diffuse peritonitis [24,25]. To our knowledge, only one study has retrospectively evaluated the association between preoperative total cholesterol levels and mortality in patients who had undergone emergency GI surgery for IAIs. The main emphasis of that study was a determination of the significance of the APACHE II scores and the MPI values as independent indicators of prognosis in patients with IAIs; preoperative hypocholesterolemia was included among the independent predictors of death [4].

In the present study, the total cholesterol levels had decreased markedly by POD 1 among both survivors and nonsurvivors. Thereafter, the total cholesterol levels in the survivors increased until POD 7, whereas those levels in the nonsurvivors remained persistently low between PODs 1 and 7. More importantly, when the total cholesterol iAUC values were compared among measurement days, the total cholesterol level on POD 7 was significantly better at predicting in-hospital mortality than measurements on other days, with an optimal cut-off value of 61 mg/dL. Recent studies have reported that severe persistent hypocholesterolemia (<50–60 mg/dL) is associated with severe illness, sepsis, and poor outcomes in postoperative patients. Furthermore, serial changes in the degree of hypocholesterolemia, over time, are useful for evaluating progressive recovery or the occurrence of complications and persistent illness in surgical patients [18,26]. A previous study of patients with sepsis and organ failure, after partial hepatectomy, found that patients did not survive if they had severe hypocholesterolemia (<58 mg/dL) for more than 6 days [13]. Thus, the present results are in accordance with the reports that document the crucial changes in cholesterol levels during the postoperative period.

Interestingly, plasma cholesterol behaves as a negative acute phase reactant [17]. It substantially decreases after trauma, acute hemorrhage, and surgery, as well as in cancer, sepsis, burn injuries, and liver dysfunction [12–15, 27–30]. Some researchers have hypothesized that low lipoprotein cholesterol levels impair the body’s ability to bind and neutralize lipopolysaccharide (LPS), allowing more LPS to be available for inducing harmful inflammation [29, 31–33]. However, analyses of LPS-lipoprotein interactions in the serum of critically ill patients have not been reported. Thus, how hypocholesterolemia might affect the innate immune responses and inflammation in these patients remains uncertain [34]. Previous studies have reported on hypocholesterolemia mechanisms, such as impaired cholesterol synthesis or hemodilution from blood loss [15,27,28,30,35–39]. Hypocholesterolemia also arises due to the combined action of multiple factors. Moreover, the degree of hypocholesterolemia may cumulatively reflect illness severity [18,38,39].

Although hypocholesterolemia is known to be a prognostic marker of increased morbidity and mortality associated with various pathological conditions [40], its impact on the in-hospital mortality of patients undergoing emergency GI surgery for diffuse peritonitis has not been previously reported. In the present study, hypocholesterolemia (<61 mg/dL) on POD 7 was significantly associated with in-hospital mortality in patients with diffuse peritonitis. The cumulative survival of patients with total cholesterol levels of ≥61 mg/dL on POD 7 was significantly better than that for those with total cholesterol levels < 61 mg/dL on POD 7, indicating that a total cholesterol level < 61 mg/dL on POD 7 is a significant predictor of in-hospital mortality.

However, to provide clinically meaningful information, a potential predictor should be measured as early as possible, before the occurrence of any clinical events, such as postoperative complications. Interestingly, the present study also showed that the total cholesterol level, measured on POD 3, significantly predicted in-hospital mortality after emergency GI surgery. This suggests that hypocholesterolemia can be used to predict early in-hospital mortality, before postoperative clinical events occur.

Multivariable analysis identified low platelet counts, newly developed sepsis, and total cholesterol < 61 mg/dL as significant predictors of in-hospital mortality after emergency GI surgery. Recent studies have reported that thrombocytopenia, after admission, is a predictor of multiple-organ failure after injury [41,42]. Moreover, thrombocytopenia is frequently accompanied by critical illness and is used as a marker of hematological organ system dysfunction. In addition, newly developed sepsis, rather than perioperative sepsis, was associated with in-hospital mortality after emergency GI surgery, in our study. This may be explained by the inclusion of repeat and late-onset sepsis, which are associated with higher mortality rates; however, the precise mechanism of this association remains unclear. Although low platelet counts and newly developed sepsis were independently associated with in-hospital mortality, we found that a total cholesterol level of <61 mg/dL on POD 7 was the strongest independent predictor of in-hospital mortality after emergency GI surgery for diffuse peritonitis. These findings suggest that severe persistent hypocholesterolemia, rather than transient hypocholesterolemia, is associated with in-hospital mortality. In contrast, a progressive increase in cholesterol is associated with patient recovery.

The present study has several limitations. First, it is a retrospective, single-center study. Second, our database does not include any information regarding nutritional factors, such as prealbumin, retinol-binding protein, or transferrin levels, nor does it include inflammatory cytokine levels, including for tumor necrosis factor-alpha, interleukin-1, or interleukin-6. These factors may affect plasma cholesterol levels, and their effects will be examined in future studies. Additionally, there was a large amount of missing data related to lactate and procalcitonin levels. For this reason, lactate and procalcitonin were excluded from the analysis. Third, we did not evaluate other lipid (e.g., high-density lipoprotein, low-density lipoprotein, and triglyceride) profiles. Cholesterol is unlikely to be the only lipid measure with an impact as a biomarker, similar to carcinoembryonic antigen and prostate-specific antigen. Therefore, cholesterol, in combination with other lipid profiles, must be validated for its intended clinical use through prospective randomized clinical trials. Fourth, fluid administration, dilution, and cholestasis were not assessed for their impacts on cholesterol levels [18]. Fifth, our database does not include information on patient histories of statin use, familial hypercholesterolemia, or types of nutritional support.

## Conclusions

This study demonstrated that severe persistent hypocholesterolemia is associated with in-hospital mortality among critically ill patients with diffuse peritonitis who undergo emergency GI surgery. Further, the total cholesterol level is a valuable biomarker for predicting outcomes during the postoperative period in these patients.

## Supporting information

S1 File STROBE statement checklist. (DOCX)

S2 File Raw data. (XLSX)
